# Heat stroke internet searches can be a new heatwave health warning surveillance indicator

**DOI:** 10.1038/srep37294

**Published:** 2016-11-21

**Authors:** Tiantian Li, Fan Ding, Qinghua Sun, Yi Zhang, Patrick L. Kinney

**Affiliations:** 1 Institute of Environmental Health and Related Product Safety, Chinese Center for Disease Control and Prevention, Beijing, China. No. 7 Panjiayuan Nanli, Chaoyang District, Beijing, 100021 China; 2 Public health Emergency Center, Chinese Center for Disease Control and Prevention, Beijing, China; 3 Mailman School of Public Health, Columbia University, New York, USA

## Abstract

The impact of major heatwave shocks on population morbidity and mortality has become an urgent public health concern. However, Current heatwave warning systems suffer from a lack of validation and an inability to provide accurate health risk warnings in a timely way. Here we conducted a correlation and linear regression analysis to test the relationship between heat stroke internet searches and heat stroke health outcomes in Shanghai, China, during the summer of 2013. We show that the resulting heatstroke index captures much of the variation in heat stroke cases and deaths. The correlation between heat stroke deaths, the search index and the incidence of heat stroke is higher than the correlation with maximum temperature. This study highlights a fast and effective heatwave health warning indicator with potential to be used throughout the world.

Over the last few decades, the impact of major heatwave shocks on population morbidity and mortality has become an urgent public health concern^1^,^2^. In the future, the frequency, intensity and duration of heatwaves is likely to increase due to climate change^3^,^4^ and there is growing recognition of the risks associated with the warming climate and the need for effective measures to anticipate and avoid its most severe consequences. Reducing the risk to health has been highlighted as a priority issue by the World Health Organization^5^,^6^. However, a lack of knowledge and preparation within the medical and healthcare systems for dealing with the effects of heatwaves is evident in most countries around the world^7^. Current heatwave warning systems suffer from a lack of validation and an inability to provide accurate health risk warnings in a timely way.

Surveillance during heatwave conditions is of critical public health concern, in order to determine whether the heatwave is associated with an increase in mortality or morbidity so that appropriate public health actions can be taken. However gathering epidemiological surveillance data using traditional methods is a labor-intensive process involving large surveys, chart reviews, prospective studies as well as data extraction and processing from databases. Current public health surveillance methods of heatwaves do not provide the information quickly enough to detect the increased number of adverse health outcomes in time to respond^7^ so its use is normally limited to retrospective analyses in order to better understand the relationship between heatwaves and health. As a result, effective surveillance systems for adverse health effects during heatwaves are very rare^8^ despite the fact that prompt surveillance information during a heatwave may be of great benefit to public health. Mortality data is widely used in surveillance for heatwave health analysis, however there is a considerable time lag between hot conditions and the reporting of deaths, which diminishes its usefulness in affecting health outcomes^7^. Furthermore, the mortality data represents the worst health outcome, which is not necessarily appropriate for use in characterizing more general public health concerns during the heatwave period^9^.

Recently, syndromic surveillance data have been used in heatwave health surveillance, such as by monitoring emergency department visits and calls to emergency or health services^10^,^11^,^12^. Even though syndromic surveillance is near real-time and forms a better representation of public health, it still does not reflect the heatwave related public health risk rapidly enough for health services to take well-timed proactive measures such as implementing a prevention plan and disseminating health warning information to ameliorate health risks^13^. There are still several reasons (such as delays in reporting from doctors, operational errors, old computer technology, etc.) that delay the reporting of health surveillance information. Making improvements in any of these areas would involve the expenditure of a large amount of human capital, administration costs and investment funds.

In current heatwave health warning systems, the prediction of possible health effects is done by modeling the relationship between temperature and health. Although this method is widely used in epidemiological studies, it has some weaknesses. Controlling for multiple confounding factors is very challenging when predicting health risks using temperature as an indicator^14^. From previous epidemiological studies, heatwaves are associated with a forward displacement of mortality known as the harvest effect. So far there is no method for accurately modeling the harvest effect during a heatwave and it is very difficult to predict health outcomes in the presence of the harvest effect^15^. Furthermore, the availability of surveillance data on health outcomes is very rare in most countries. Most of the temperature and health outcomes are studied using mortality data while few use morbidity data, but the former is not well suited for representing general population health and it still leaves a high level of uncertainty in health risk prediction during heatwaves. Nowadays, the increasing availability of datasets from sources such as social media posts, search engine queries and other internet data have shown the potential for analyzing patterns, trends and social phenomena in a variety of domains including finance^16^,^17^, science^18^, tourism^19^,^20^ and health^21^,^22^,^23^,^24^. In this study, we conducted a correlation and linear regression analysis to test the relationship between heat stroke internet searches and heat stroke health outcomes in Shanghai, China, during the summer of 2013. We also developed and tested a new preliminary surveillance proxy during the heatwave period aimed at addressing the shortcomings of current heatwave health warning surveillance.

## Results

**Figure 1:**
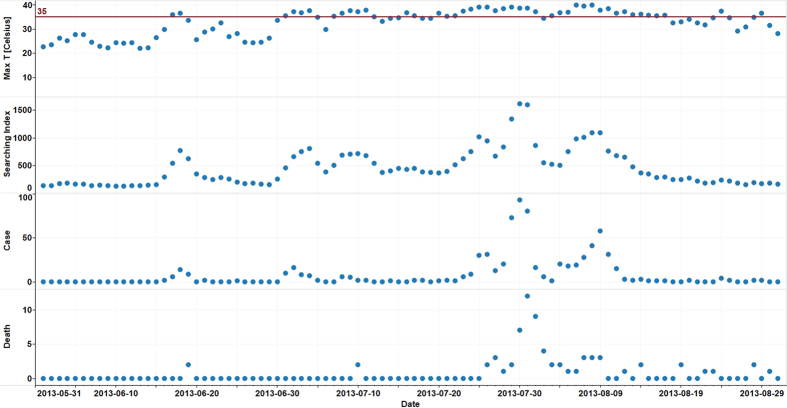
Time series plots of maximum temperature, heat stroke internet searching index, heat stroke surveillance data in the summer of 2013, Shanghai.

**Figure 2:**
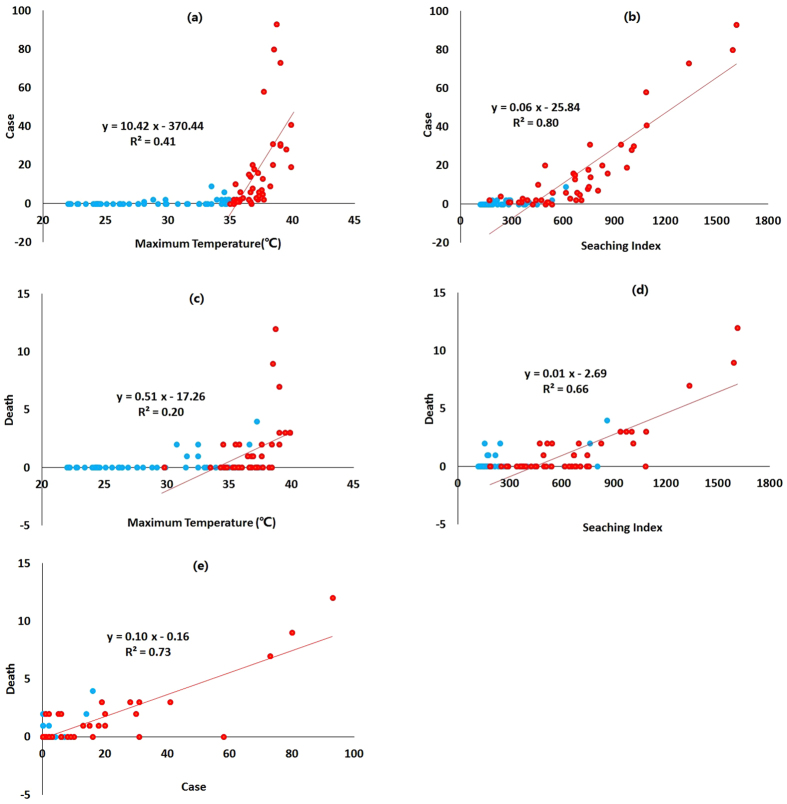
plots of variables for the whole summer period and linear regression model for temperatures above 35 °C. (**a**) Heat stroke cases ~ Maximum Temperature (same day); (**b**) Heat stroke cases ~ Searching index (same day); (**c**) Heat stroke deaths ~ Maximum Temperature (lag 1 day); (**d**) Heat stroke deaths ~ Search index (lag 1 day); (**e**) Heat stroke deaths ~ Heat stroke cases (lag 1 day). Red dots represent occasions when the maximum temperature was ≥35 °C, and the red lines represent the linear regression line for those occasions.

Our analysis is based upon 92 days of data from June 1 to August 31, 2013. The mean daily maximum temperature during this period was 32.9 °C, with a maximum temperature of 39.9 °C. In China, a heatwave is defined as three consecutive days in which the maximum temperature is at or above 35 °C^25^. There were 23 days in which the temperature reached 35 °C, which led to four heatwaves, two of which lasted more than 10 days. The longest heatwave continued for 15 days. There were on average 8.0 heat stroke cases and 0.8 heat stroke deaths per day over the summer period. The maximum number of heat stroke cases in one day was 93 and the most deaths in one day was 12. Table 1 shows that the mean daily heat stroke internet search index, as published by the Baidu search engine was 450.4 and the maximum index was 1165.0. Figure 1 plots the temperature, internet search index, number of cases of heat stroke and number of deaths from heat stroke for the summer period. It can be seen that the heat stroke index captures the variation of heat stroke cases and deaths very well. Whereas, the maximum temperature time series plots do not appear to correlate as well with heat stroke cases and deaths. Table S1 shows this in quantitative terms in that the correlation between the number of heat stroke cases and the search index was higher than the correlation between maximum temperature and heat stroke cases. Figure 2(a and b) show that the search index, with an R^2^ of 0.80, is a better predictor of heat stroke cases than the maximum temperature. Table S3 shows that the correlation between heat stroke cases and deaths with the search index was higher than its correlation with maximum temperature during the overall study period.

The same day correlation was the highest for both the search index and maximum temperature. When only using the data for which the maximum temperature is at or above 35 °C, we observed higher values for the same day correlation, in particular, the same day correlation between heat stroke cases and search index had a value of 0.89 (p < 0.01) (Table S2).

The lag 1 day correlation was highest among all the lag day variations. When only taking data where there was a maximum temperature at or above 35 °C, the lag 1 day correlation values were larger, with a correlation coefficient of 0.81 (p < 0.01) for the heat stroke search index and 0.86 (p < 0.01) for heat stroke cases (Table S4). Figure 2(c–e) show that the 1 day lagged search index and 1 day lagged heat stroke cases were both good indicators for predicting the number of heat stroke deaths with an R^2^ of 0.66 and 0.73 respectively.

**Figure 3:**
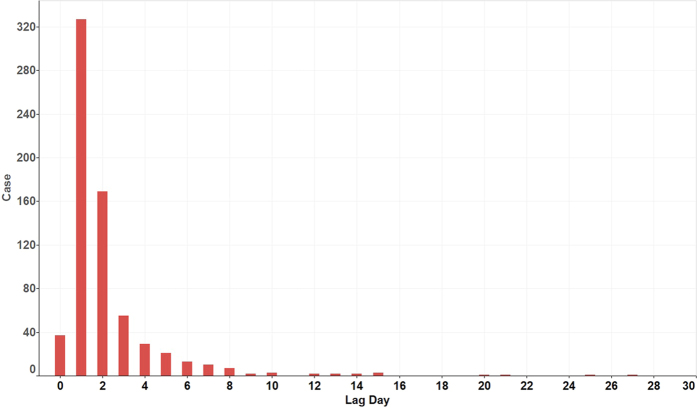
Reporting lag days of heat stroke case registration.

Figure 3 shows the number of days for the delay of heat stroke case registration. The surveillance data we used in Shanghai for this study is nearly real time, collecting the heat stroke cases directly from hospital and transferred by website. However, only 5% of cases reported on the same day. Hence, it is very difficult to identify the heat wave health epidemic rapidly using present surveillance data.

## Discussion

Our study showed that the relationship between heat stroke internet searches and health outcomes is much higher than that between temperature and health outcomes. The heat stroke internet searches had better predictive ability for health risk than temperature during the heatwave. The temperature and health epidemiological studies show the lag effect of mortality, is in agreement with previous studies^15^,^26^. Our study also shows a significant correlation between heat stroke cases and heat stroke deaths. This indicates that morbidity surveillance data could be a good indicator for mortality during a heatwave. Hence, the identification and prevention of morbidity during a heatwave can be expected to decrease the mortality rate.

A new method of syndromic surveillance has emerged^8^,^22^,^27^,^28^ which is known as web-based keyword searching. This has been shown to be a feasible surveillance method for influenza, kidney stone disease, dengue and other conditions. Heat stroke is a widely known effect of heatwaves and is associated with easily recognizable heatwave related syndromic keywords in search engines. The consistency of both factors in our study is much higher than in previous studies of other diseases and internet keyword searching^8^,^28^,^29^,^30^. Therefore, the heat stroke internet search is well suited for use as a new heatwave surveillance health outcome proxy. In 2013, the internet search based Google Flu Trends was predicting more than double the proportion of doctor visits for influenza like illnesses than the Center for Disease Control and Prevention (CDC)^31^. Lazer *et al.* explored the “big data hubris” and algorithm dynamics that contributed to Google Flu Trends’ mistakes^31^. The problem was mainly due to a mismatch between number of searches, about 50 million, and the small volume of surveillance data of 1152 cases^31^. In our study, the number of searches is about 450 per day and the number of heat stroke cases and deaths is about 8 and 1 per day respectively. The search index is at most several hundred times the number of health outcomes, which is much smaller than that of Google Flu Trends. Google Flu Trends also used an algorithm to capture the dynamics of the cases^31^. However, in our analysis, the search index value was directly compared with heat stroke health outcomes, which capture the variation in cases and deaths very well. Therefore, heat stroke searches can be expected to largely avoid the two issues that led to Google Flu Trends prediction errors. A recent study of Google Flu Trends showed that using the aggregate frequency of selected queries as the only predictor could lead the prediction errors^32^. We also aggregated all queries about “heat stroke” in a single predictor, hence our indicator may also be prone to similar errors. However, the purpose of this study is not to build a predictive model for heat wave related health outcomes. We identify this potential weakness for further studies to address.

Recently the use of web based searching for disease identification has been gaining interest and mining the web is a valuable new direction to quickly identify diseases and epidemics^8^,^24^,^29^,^33^. Our study is the first to show that web based searching could be useful for predicting risks to health during heatwaves and provides a new heatwave health warning system. This offers the prospect that stakeholders could recognize the heatwave health epidemic in a timely and cost effective way, which could translate into a practical and rapid health protection response.

It is however also important to note the limitations of this new syndromic surveillance tool. Demographic information on users is not available. Therefore, it is not possible to identify the most vulnerable population during the heatwave. In addition, the precise reason for users searching for terms is not clear. The keyword “heat stroke” is not the only one that could be used and the effectiveness of alternative keywords will be investigated in further research. There are also many confounding factors that could not be modeled. It is noteworthy that publicity due to a health awareness campaign or items from the media can affect internet searches^23^. This could be addressed using a web-based database to monitor news items, which could be used to adjust the model. In Shanghai about 30% of people do not have access to the internet in 2013^34^ and it is possible that this group includes a disproportionate number of older people who are especially vulnerable to the effects of heatwaves. This may be another source of uncertainty in using internet searches for heatwave surveillance data. As a result of data limitations, we have only been able to test the internet searches during heatwaves in one location during one summer period. Further testing of the relationship between “heat stroke” internet searches and health outcomes during heatwaves in other locations in different heatwave periods is anticipated in the future.

Internet searches are easily accessible and economical, which could be of benefit for the early warning of health risks and taking preventive measures during the heat wave period. A unique strength of internet searches is the immediacy of access to the data, which provides the basis for an alternative real-time health surveillance system. Compared with traditional surveillance data, internet searches could be used for recognizing the onset of epidemics more quickly and producing real-time health warnings during heatwaves. Heatwaves are a global public health concern and will be more frequent and severe in a changing climate. However, health surveillance during heatwaves and heatwave health warning systems are very rare around the world. Our study shows that heat stroke internet searching could form a new tool for confronting the challenge of heatwaves worldwide. Nowadays, with the rapid development of the internet, web searching will become more accurate and representative of the whole population. It could be used in different areas, especially in regions with no health surveillance records.

## Methods

The study period was June 1 to Aug 30, 2013. The data on heat stroke cases and deaths in Shanghai, China were obtained from the heat stroke surveillance register of the Chinese Center for Disease Control and Prevention. Temperature data for the Shanghai Xujiahui station was obtained from the Chinese Meteorological Data Sharing Service System. Daily index values in web searches containing the key word “heat stroke” within Shanghai were collected from the Baidu index website. “Heat stroke” corresponds to an exclusive technical term in Chinese, which is “
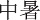
”. We used the Chinese characters “
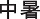
” as the key word to collect the corresponding Baidu index values. The Baidu index reflects keyword search volume, which was developed by Baidu Inc. It uses the search queries on Baidu web searches to calculate the Baidu index, and represents the search frequency of certain keywords on Baidu web searches based on the actual searching behavior of its users^35^. In 2013, Baidu occupied more than 80% of the Chinese search engine market^36^, making it better suited than Google trends to model the search behavior of the Chinese Population. In recent years, the Baidu index has been used to forecast infectious diseases, tourist volumes, economic indicators etc.^16^,19,^37^. Shanghai is one of the fastest developing cities in China, the number of internet users was 16.83 million with an internet penetration rate of 70.7% in 2013^34^. Therefore, the Baidu index provides a good representation of searching behavior in Shanghai. Correlation analysis and linear regression analysis were performed in this study using R 3.2.2.

## Additional Information

**How to cite this article**: Li, T. *et al.* Heat stroke internet searches can be a new heatwave health warning surveillance indicator. *Sci. Rep.* **6**, 37294; doi: 10.1038/srep37294 (2016).

**Publisher’s note**: Springer Nature remains neutral with regard to jurisdictional claims in published maps and institutional affiliations.

## Supplementary Material

Supplementary Information

## Figures and Tables

**Table 1:** Descriptive statistical analysis of data from June 1 to Aug 31, 2013, Shanghai.

	N	Mean	SD	Min	Max
Maximum Temperature	92	32.9	5.2	22.0	39.9
Searching Index	92	450.4	329.3	116.0	1165.0
Heat Stroke Cases	92	8.0	17.0	0	93.0
Heat Stroke Deaths	92	0.8	1.9	0	12.0
