# Supplementary material for: Heat stroke internet searches can be a new heatwave health warning surveillance indicator
Source: Sci Rep. 2016 Nov 21;6:37294. doi: 10.1038/srep37294 (PMC5116664; doi:10.1038/srep37294)
Supplement: Supplementary Information [file srep37294-s1.pdf]

Supplementary Information

Heat stroke internet searches can be a new heatwave health warning surveillance indicator

Tiantian Li[1*], Fan Ding[2], Qinghua Sun[1], Yi Zhang[1], Patrick L. Kinney[3]

[1]Institute of Environmental Health and Related Product Safety, Chinese Center for Disease Control and Prevention, Beijing, China. No. 7 Panjiayuan Nanli, Chaoyang District, Beijing, 100021 China.

[2]Public health Emergency Center, Chinese Center for Disease Control and Prevention, Beijing, China.

[3]Mailman School of Public Health, Columbia University, New York, USA.

Tiantian Li* E-mail: tiantianli@gmail.com Telephone: 008613671359855

Fan Ding E-mail: dingfan@chinacdc.cn

Qinghua Sun E-mail: qhsunpku@gmail.com

Yi Zhang E-mail: zhangyimedical@126.com

Patrick L. Kinney E-mail: plk3@cumc.columbia.edu

*Corresponding author

Table S1 Pearson correlations for lag 0-4 heat stroke internet searching index and lag 0-4 maximum temperature with heat stroke case data in the summer of 2013, Shanghai

Lag days	Searching index	Maximum temperature
0	0.86**	0.47**
1	0.73**	0.46**
2	0.51**	0.40**
3	0.37**	0.35**
4	0.34**	0.31**

**Correlation is significant at the 0.01 level (2-tailed)

*Correlation is significant at the 0.05 level (2-tailed)

Table S2 Pearson correlations for lag 0-4 heat stroke internet searching index and lag 0-4 maximum temperature with heat stroke case data in high temperature days (≥ 35°C) of 2013, Shanghai

Lag days	Searching index	Maximum temperature
0	0.89**	0.64**
1	0.72**	0.54**
2	0.48**	0.39**
3	0.27	0.29
4	0.22	0.19

**Correlation is significant at the 0.01 level (2-tailed)

*Correlation is significant at the 0.05 level (2-tailed)

Table S3 Pearson correlations for lag 0-4 heat stroke internet searching index, lag 0-4 maximum temperature and lag 0-4 heat stroke case data with heat stroke deaths in the summer of 2013, Shanghai

Lag days	Searching index	Maximum temperature	Heat stroke cases
0	0.63**	0.34**	0.69**
1	0.73**	0.37**	0.84**
2	0.65**	0.37**	0.73**
3	0.47**	0.35**	0.48**
4	0.36**	0.32**	0.33**

**Correlation is significant at the 0.01 level (2-tailed)

*Correlation is significant at the 0.05 level (2-tailed)

Table S4 Pearson correlations for lag 0-4 heat stroke internet searching index, lag 0-4 maximum temperature and lag 0-4 heat stroke case data with heat stroke deaths in high temperature days (≥ 35°C) of 2013, Shanghai

Lag days	Searching index	Maximum temperature	Heat stroke cases
0	0.67**	0.38*	0.68**
1	0.81**	0.44**	0.86**
2	0.71**	0.42**	0.73**
3	0.44**	0.35*	0.42**
4	0.27	0.28	0.22

**Correlation is significant at the 0.01 level (2-tailed)

*Correlation is significant at the 0.05 level (2-tailed)
